# Trpc6 Promotes Doxorubicin-Induced Cardiomyopathy in Male Mice With Pleiotropic Differences Between Males and Females

**DOI:** 10.3389/fcvm.2021.757784

**Published:** 2022-01-13

**Authors:** Nadine Norton, Katelyn A. Bruno, Damian N. Di Florio, Emily R. Whelan, Anneliese R. Hill, Andrea Carolina Morales-Lara, Anna A. Mease, John M. Sousou, Jose A. Malavet, Lauren E. Dorn, Gary R. Salomon, Logan P. Macomb, Sami Khatib, Zacharias P. Anastasiadis, Brian M. Necela, Molly M. McGuire, Presley G. Giresi, Archana Kotha, Danielle J. Beetler, Raegan M. Weil, Carolyn K. Landolfo, DeLisa Fairweather

**Affiliations:** ^1^Department of Cancer Biology, Mayo Clinic, Jacksonville, FL, United States; ^2^Department of Cardiovascular Medicine, Mayo Clinic, Jacksonville, FL, United States; ^3^Center of Clinical and Translational Science, Mayo Clinic, Jacksonville, FL, United States

**Keywords:** cardiotoxicity, anthracycline, heart failure, sex differences, ion channel, TRPC

## Abstract

**Background:** Doxorubicin is a widely used and effective chemotherapy, but the major limiting side effect is cardiomyopathy which in some patients leads to congestive heart failure. Genetic variants in *TRPC6* have been associated with the development of doxorubicin-induced cardiotoxicity, suggesting that TRPC6 may be a therapeutic target for cardioprotection in cancer patients.

**Methods:** Assessment of *Trpc6* deficiency to prevent doxorubicin-induced cardiac damage and function was conducted in male and female B6.129 and Trpc6 knock-out mice. Mice were treated with doxorubicin intraperitoneally every other day for a total of 6 injections (4 mg/kg/dose, cumulative dose 24 mg/kg). Cardiac damage was measured in heart sections by quantification of vacuolation and fibrosis, and in heart tissue by gene expression of *Tnni3* and *Myh7*. Cardiac function was determined by echocardiography.

**Results:** When treated with doxorubicin, male *Trpc6*-deficient mice showed improvement in markers of cardiac damage with significantly reduced vacuolation, fibrosis and *Myh7* expression and increased *Tnni3* expression in the heart compared to wild-type controls. Similarly, male *Trpc6*-deficient mice treated with doxorubicin had improved LVEF, fractional shortening, cardiac output and stroke volume. Female mice were less susceptible to doxorubicin-induced cardiac damage and functional changes than males, but *Trpc6*-deficient females had improved vacuolation with doxorubicin treatment. Sex differences were observed in wild-type and *Trpc6*-deficient mice in body-weight and expression of *Trpc1, Trpc3* and *Rcan1* in response to doxorubicin.

**Conclusions:** Trpc6 promotes cardiac damage following treatment with doxorubicin resulting in cardiomyopathy in male mice. Female mice are less susceptible to cardiotoxicity with more robust ability to modulate other Trpc channels and Rcan1 expression.

## Introduction

Doxorubicin is a widely used and effective chemotherapy agent for multiple adult and pediatric cancers. However, a potential side effect is cumulative, dose-related, progressive myocardial damage that can lead to congestive heart failure (CHF), even several years after completion of treatment (1–6). The mechanisms of cardiotoxicity leading to cardiomyopathy are likely complex including generation of reactive oxygen species (ROS) and iron (7), doxorubicin binding to topoisomerases (8), impaired mitochondrial function (9), disruption of calcium homeostasis (10–12), up-regulation of death receptors (13), and up-regulation of the potent vasoconstrictor endothelin 1 which causes fibrosis and the generation of ROS (14).

ASCO guidelines for monitoring and preventing cardiac dysfunction after doxorubicin therapy state that currently there is not sufficient evidence to recommend any single heart failure medication such as angiotensin-converting enzyme (ACE) inhibitors or beta blockers to improve function (15). To date, the only FDA-approved cardioprotective drug for doxorubicin-induced cardiomyopathy is the iron chelating agent, Dexrazoxane, which is thought to deplete topoisomerase IIb (16, 17) and prevent mitochondrial iron-catalyzed ROS damage (7). However, for a therapy to be useful in mediating cardioprotection it is important that it does not counteract the anti-tumor effect of the chemotherapy agent, and there are concerns that Dexrazoxane may interfere with the antitumor efficacy of doxorubicin (18). To date, alternative iron chelators have yielded negative or mixed outcomes (7) indicating that there is a need to find alternative strategies for cardioprotection from anthracyclines.

Given the multiple, complex mechanisms of doxorubicin-induced cardiotoxicity and the variability in patient cardiovascular outcome, we previously used a genome-wide approach to identify genetic variants that were associated with doxorubicin-induced decline in left ventricular ejection fraction (LVEF) (19). That study identified transient receptor potential cation channel subunit 6 (*TRPC6)* as a potential risk locus for doxorubicin-induced cardiomyopathy in patients with breast cancer (19). In a follow-up study from our group using 984 patients from the Mayo Clinic Biobank, we replicated the association of toxicity, specifically with the outcome of doxorubicin-induced congestive heart failure (CHF) (20).

TRPC1-7 channels are an important group of calcium permeable ion channels that induce changes in cardiac function in response to cardiac strain and/or disease (21). Different missense mutations in *TRPC6* have been shown to result in excess calcium influx, largely by gain-of-function mutations (22), leading to the hypothesis that individuals carrying *TRPC6* variants could be at increased risk of doxorubicin-induced cardiotoxicity and cardiomyopathy and perhaps be candidates for TRPC inhibition as a cardioprotective strategy. A number of studies have demonstrated the potential of TRPC1, 3 and/or 6 channels as therapeutic targets for heart failure, predominantly using *in vivo* models of pressure overload in male mice (23–25). We previously published that pre-treatment of male mice with a TRPC6 inhibitor GsMTx-4 significantly reduced fibrosis and improved LVEF and cardiac strain in mice given doxorubicin (20). In this study, we hypothesized that genetic deficiency of *Trpc6* would decrease cardiotoxicity and cardiomyopathy in male and female mice given doxorubicin.

## Materials and Methods

### Chemotherapy Agent

Doxorubicin was purchased from Selleckchem (Houston, TX) in powder form (25 mg) and dissolved in sterile water as 1.25 mL aliquots to a concentration of 20 mg/mL and stored at 4°C. For injections, the 20 mg/mL stock solution was diluted in sterile saline to a final concentration of 1 mg/mL.

### Animal Model

Animal protocols were performed according to NIH guidelines with approval from the Institutional Animal Care and Use Committee, Environmental Health and Occupational Safety Committee and the Biosafety Committee at Mayo Clinic. Mice were bred and maintained under pathogen-free conditions in the animal facility at the Mayo Clinic, fed standard chow and water *ad libitum*, and housed in animal rooms where the temperature was monitored. Breeding pairs of B6.129 wild-type (WT) (Cat#101045) and B6.129 Trpc6 whole body knock-out (KO) mice (26) (Cat#37345) were obtained from the Jackson Laboratory (Bar Harbor, ME). Male and female WT and Trpc6 KO mice (8–10 weeks old), ten mice per group, received either 100 μL intraperitoneally (ip) of control sterile saline or 4 mg/kg/dose doxorubicin for a cumulative dose of 24 mg/kg on days 1, 3, 5, 7, 9, 11 according to (20). Results were confirmed by repeating each experiment. Hearts were evaluated for cardiac function using echocardiography and tissues collected on day 14 and 21.

### Echocardiography

Cardiac function was performed by transthoracic echocardiogram using a Visual Sonic Vevo 2100 with a 55-megahertz (MHz) transducer (Bothell, WA). Echocardiography was performed on living male and female animals under isoflurane inhalation at day 14 and 21 as per our previous publications (20, 27–30).

### Histology

Mouse hearts were cut longitudinally, fixed in 10% phosphate-buffered formalin, and embedded in paraffin for histological analysis. Five-micron-thick sections were stained with hematoxylin and eosin to detect vacuolation or trichrome blue to detect fibrosis. Vacuolation and fibrosis were calculated as the number of grids with vacuoles or fibrosis, respectively, compared to the total number of grids in the heart section using an eyepiece grid with a 2x objective lens (20x magnification) and converted to a percentage, as previously (31, 32). Sections were scored by two individuals blinded to experimental group.

### RNA Extraction

At harvest, half of the heart was collected and stored at −80°C for RNA isolation. Hearts were homogenized and lysed using Tissuelyser (Qiagen) with 7 mm stainless steel beads in RTL buffer with 0.5% DX buffer to reduce foam (Hilden, Germany). The homogenate was then placed in an automated RNA isolation and purification instrument, QIAcube, with reagents for RNase Easy Fibrous Mini Kit including a DNase and Proteinase K step (Qiagen #74704). RNA was eluted into 30 μL. If the heart had been divided in the earlier step, the eluted RNA was pooled prior to being aliquoted. RNA quantification was determined in μg/μL using NanoDrop (Thermo Scientific, Waltham, MA).

### Quantitative PCR

Two-step quantitative reverse transcriptase-mediated real-time PCR (qPCR) was used to measure abundance of individual mRNAs. Total RNA from mouse hearts was assessed by quantitative real time (qRT)-PCR using Assay-on-Demand primers and probe sets and the ABI 7000 Taqman System from Applied Biosystems (Foster City, CA) after RNA was converted to cDNA using a High Capacity cDNA Reverse Transcriptase Kit (Applied Biosystems), and qPCR reactions were performed in triplicate with 100 ng of cDNA and the TaqMan Universal PCR master mix (Applied Biosystems), as previously described (28, 29). The following primer/probe sets were purchased from Applied Biosystems: Trpc1 (Mm00441975_m1), Trpc3 (Mm00444690_m1), Trpc6 (Mm01176083_m1), Myh7 (Mm00600555_m1), Tnni3 (Mm00437164_m1) and Rcan1 (Mm01213406_m1). Amplification data were collected with an Applied Biosystems ViiA7 detector and analyzed with ViiA7 v 1.2.4 software (Life Technologies). Data were normalized to the endogenous control *Polr2a* (Mm00839502_m1) (33) and mRNA abundance was calculated using the ΔΔCT method and displayed as fold change (FC) (34).

### TUNEL Assay

Hearts were fixed in 10% buffered formalin for 48 h and transferred to containers of PBS prior to paraffin embedding and mounting on slides. TUNEL Assay was performed using the Click-iT Plus TUNEL Assay for *in situ* Apoptosis Detection on the Alexa 647 (ThermoFisher, Cat: C10619). Slides were deparaffinized per manufacturer recommendations and steamed for 30 min prior to permeabilizing with Proteinase K. Tissue autofluorescence was quenched with Vector TrueVIEW Autofluorescence Quenching Kit (Vector Laboratories, Cat: SP-8400-15). Heart sections were incubated with TdT Reaction Buffer for 20 min at 37°C prior to performing the TdT Reaction for 60 min at 37°C. TUNEL reaction was performed for 45 min at 37°C. Nuclei were counter-stained with Hoechst 33342 (ThermoFisher, Cat: H21492) and then mounted with Vectashield Antifade Mounting Medium (Vector Labs, Cat: H-1000-10). After drying for 48 h, slides were scanned with a Panoramic 250 fluorescent slide scanner (3DHISTECH). The ventricles of heart sections were selected and annotated in CaseViewer (3DHISTECH). TUNEL positivity was determined in QuantCenter (3DHISTECH) using cell quant with the following parameters: Channel Matching – default; Detection – nuclei selected for both the DAPI and Cy5 channels; Nuclei – contrast set to 35, other settings were default; Cytoplasm – n/a; Membrane – n/a; Scoring – object selected was nuclei and channel selected was Cy5. These parameters allowed for identification of all nuclei and then determination of the frequency of TUNEL/Cy5 positivity where the aggregate score of Medium and Strong Positive Nuclei = TUNEL positive.

### Statistical Analysis

Statistical analyses were performed in GraphPad Prism 9.0.1. Differences between two groups were tested by unpaired 2-tailed Student's *t*-test. Differences between more than two groups were tested by one-way ANOVA followed by Tukey's or Holm-Sidak's multiple comparison tests. Differences between groups over time were compared by two-way ANOVA. Survival curves were analyzed by log-rank (Mantel-Cox) test. Data are expressed as mean ±SEM. A value of *p* < 0.05 was considered significant.

## Results

### Trpc6 Deficiency Improves Survival in Male Mice Following Doxorubicin Treatment

**Figure 1:**
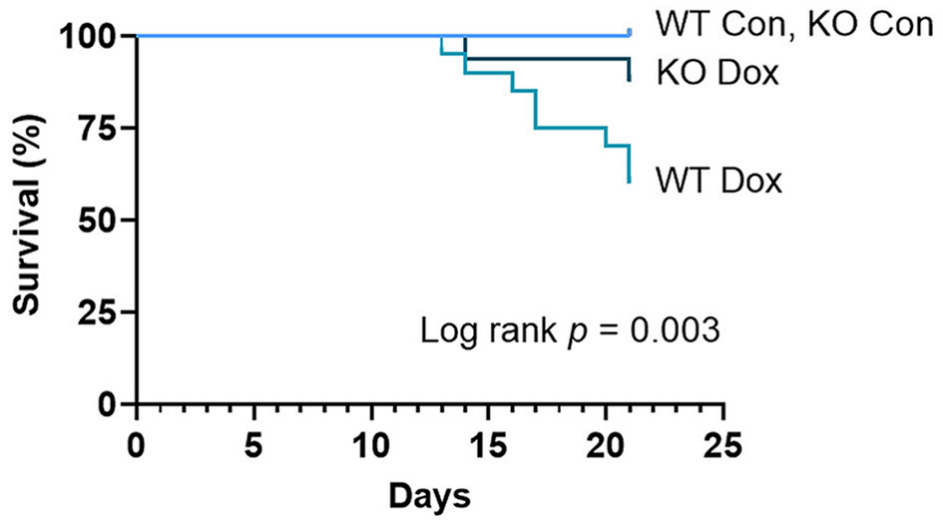
Trpc6 deficiency improves survival in male mice treated with doxorubicin. Male wild-type (WT) and *Trpc6*-deficient (KO) mice were treated with 100 mL saline (CON) or 4 mg/kg doxorubicin (DOX) on days 1, 3, 5, 7, 9, 11 for a cumulative dose of 24 mg/kg. Two separate experiments of 10 mice/group were combined (*n* = 20/group) and analyzed by log-rank (Mantel-Cox) test.

Shortly after the accumulative dose of doxorubicin was achieved at day 11 post inoculation, male wild-type and *Trpc6*-deficient mice began to die, although the specific cause of death was not ascertained (Figure 1). Deficiency in *Trpc6* improved survival after doxorubicin treatment in males, (*p* = 0.003, Figure 1). These findings suggest that *Trpc6* contributes to mortality following doxorubicin therapy in male mice.

### Trpc6 Deficiency Improves Doxorubicin-Induced Body Weight Loss in Males

**Figure 2:**
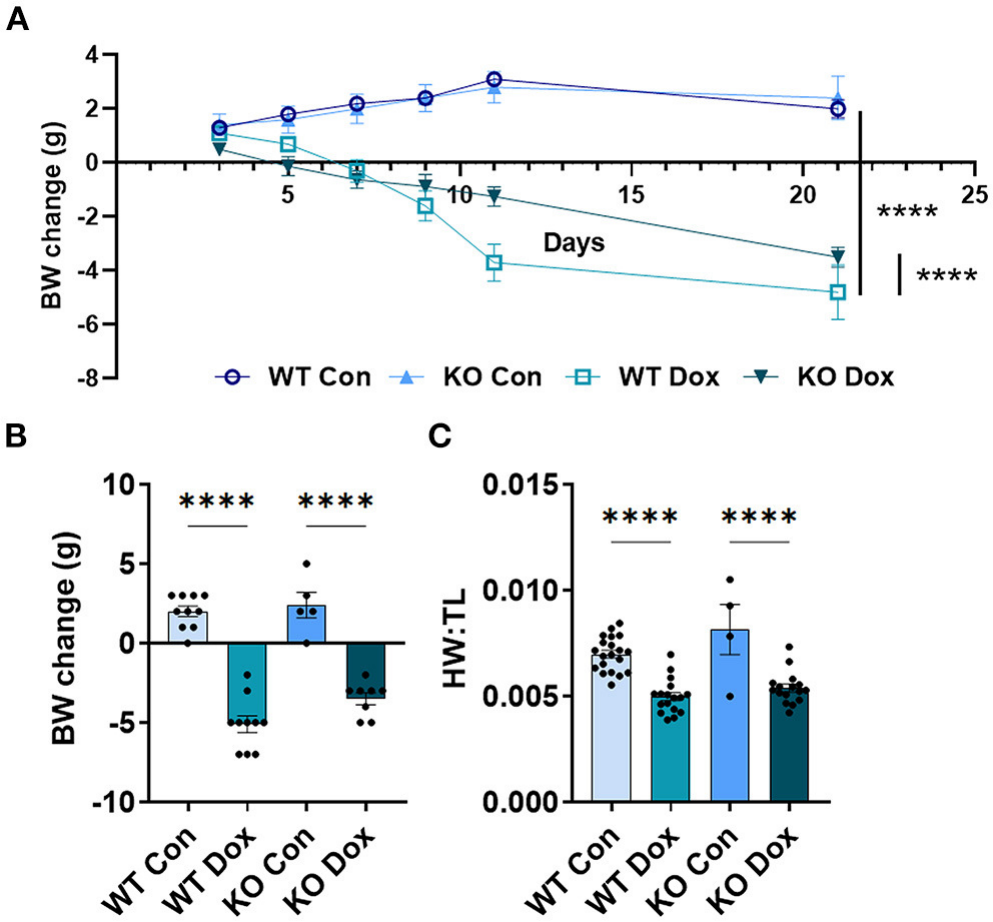
*Trpc6*-deficiency reduces doxorubicin-induced body weight loss in males. Male wild-type (WT) and *Trpc6*-deficient (KO) mice were treated with 100 mL saline (CON) or 4 mg/kg doxorubicin (DOX) on days 1, 3, 5, 7, 9, 11 for a cumulative dose of 24 mg/kg. **(A)** Change in body-weight (BW) from baseline in grams (g) over time. Data were analyzed by two-way ANOVA. **(B)** Change in body weight (BW) comparing baseline to day 21. **(C)** Ratio of heart weight to tibia length (HW:TL) at day 21. ****p < 0.0001. Data shown as mean +/- SEM using one-way ANOVA with Tukey's multiple comparisons test with 4–20 mice/ group.

Mice were weighed immediately prior to each injection of doxorubicin to ensure the correct dose was used (approximately 4mg/kg per dose). As expected, both wild-type and *Trpc6*-deficient males treated with doxorubicin progressively lost body-weight relative to control mice (*p* < 0.0001, Figure 2A), while wild-type and *Trpc6*-deficient control males maintained their weight over the duration of the experiment (*p* = 0.724, Figure 2A). The loss in weight for wild-type and Trpc6-deficient mice treated with doxorubicin was observed at day 21 (*p* < 0.0001, Figure 2B). However, *Trpc6*-deficient mice treated with doxorubicin lost less weight than wild-type mice treated with doxorubicin over the duration of the experiment (*p* < 0.0001, Figure 2A), suggesting that *Trpc6* worsens the effects of doxorubicin.

In mice that survived to day 21, we also determined the heart-weight to tibia length (HW:TL) ratio. An elevated HW:TL indicates cardiac hypertrophy. Instead, we found that doxorubicin treatment caused a reduction in HW:TL in wild-type and *Trpc6*-deficient males (*p* < 0.001, Figure 2C), indicating cardiac damage, that was not recovered by *Trpc6* deficiency (*p* = 0.64, Figure 2C). Thus, *Trpc6* contributes to loss of body weight due to doxorubicin treatment but does not alter heart weight in male mice.

### Trpc6 Deficiency Improves Cardiac Damage and Function at Day 21 in Male Mice Treated With Doxorubicin

**Figure 3:**
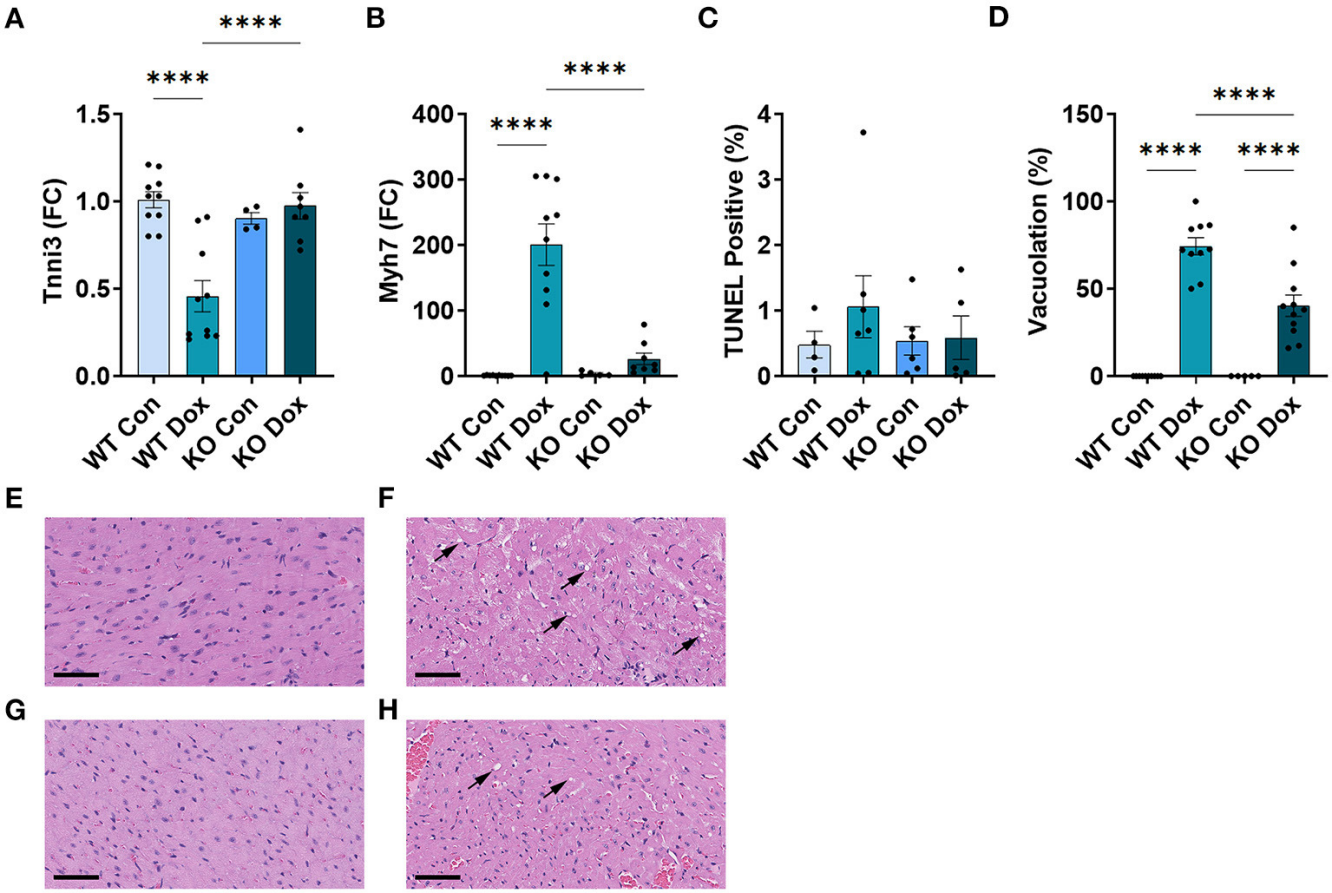
*Trpc6*-deficiency reduces cardiac damage following doxorubicin in males. Male wild-type (WT) and *Trpc6*-deficient (KO) mice were treated with 100 mL saline (CON) or 4 mg/kg doxorubicin (DOX) on days 1, 3, 5, 7, 9, 11 for a cumulative dose of 24 mg/kg. **(A)** Cardiac troponin (*Tnni3*) or **(B)** myosin heavy chain 7 (*Myh7*) gene expression shown as a fold change (FC) relative to WT Control. **(C)** TUNEL Positive (%). **(D)** Percent vacuolation. ****p < 0.0001. Data shown as mean +/- SEM using one-way ANOVA with Tukey's multiple comparisons test with 4–10 mice/ group. Hematoxylin and eosin **(**H&E) staining of representative heart sections from **(E)** male wild-type (WT) mice treated with saline (Con), **(F)** male wild-type (WT) mice treated with doxorubicin (Dox), **(G)** *Trpc6*-deficient (KO) mice treated with saline, or **(H)** *Trpc6*-deficient (KO) mice treated with Dox showing vacuoles in black arrows. Magnification 400x. Scale bars are 60 μm.

We next examined gene expression of two known biomarkers of heart damage, cardiac troponin (*Tnni3*) and myosin heavy chain 7 (*Myh7*, also known as myosin heavy chain beta), in male mice at day 21. Both *Tnni3* and *Myh7* gene expression was significantly different between groups by ANOVA (*p* < 0.0001 and *p* < 0.0001, respectively, Figures 3A,B). *Tnni3* expression in the heart of wild-type mice was significantly reduced by doxorubicin treatment compared to saline controls, *p* < 0.0001, and the reduction was almost completely reversed by *Trpc6* deficiency, (*p* < 0.0001, Figure 3A), indicating that *Trpc6* promotes cardiac damage. *Myh7* expression, which is known to increase in failing human (35, 36) and mouse hearts (37, 38), increased significantly in male wild-type mice treated with doxorubicin, *p* < 0.0001, and was also reversed by *Trpc6* deficiency, (*p* < 0.0001, Figure 3B), indicating that *Trpc6* promotes cardiac damage. The gene expression levels of *Tnni3* and *Myh7* were very similar between wild-type and *Trpc6*-deficient saline control males indicating that there was no apparent underlying difference in cardiac damage between the two mouse strains. Together, these data show that *Trpc6* worsens cardiac damage in response to doxorubicin.

**Figure 4:**
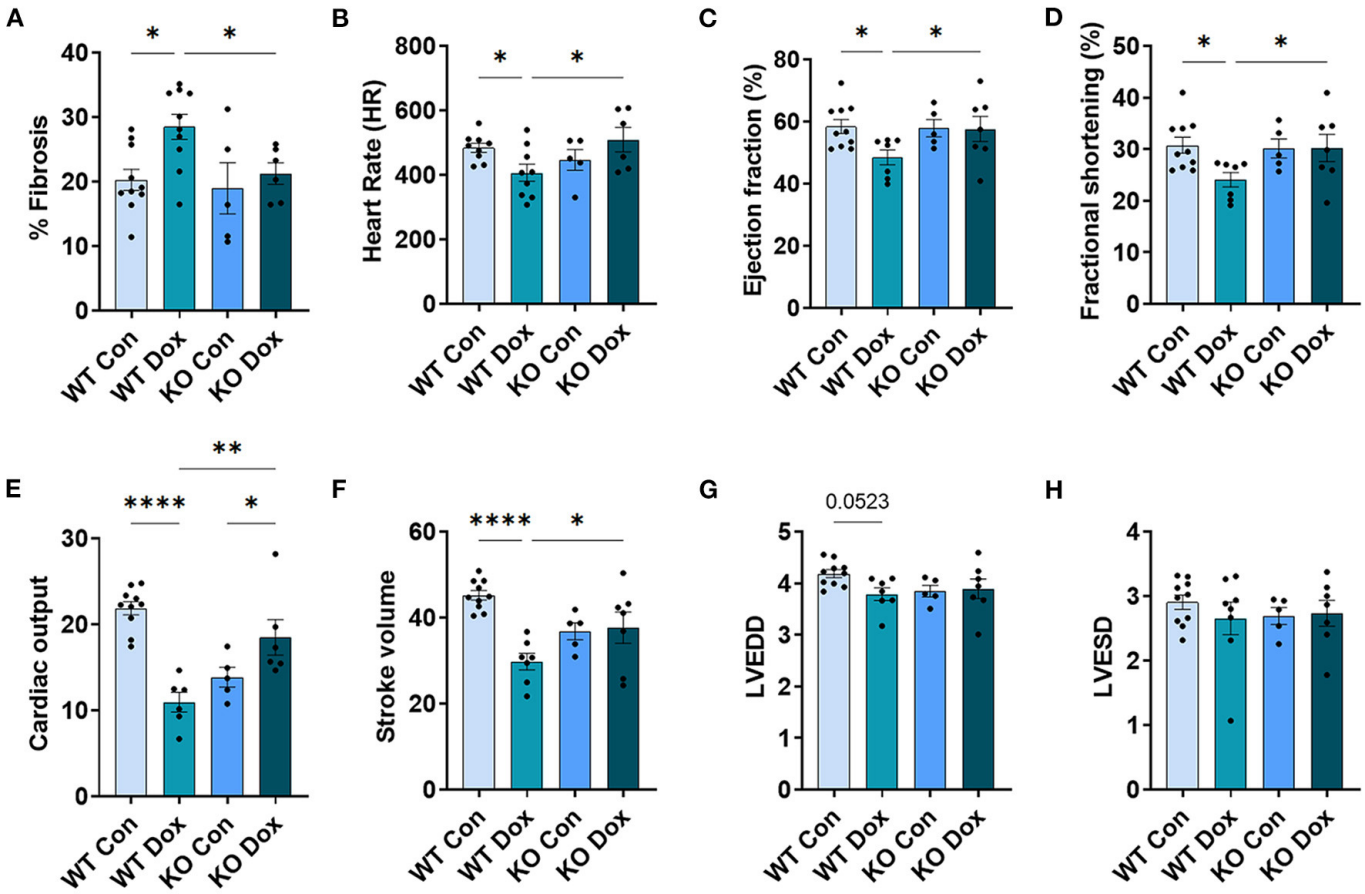
*Trpc6*-deficiency improves cardiac function assessed using echocardiography in males treated with doxorubicin. Male wild-type (WT) and *Trpc6*-deficient (KO) mice were treated with 100 mL saline (CON) or 4 mg/kg doxorubicin (DOX) on days 1, 3, 5, 7, 9, 11 for a cumulative dose of 24 mg/kg. Trichrome blue staining was performed at day 21 to assess **(A)** % fibrosis. Echocardiogram was performed at day 21 to assess **(B)** heart rate (HR), **(C)** % left ventricular (LV) ejection fraction, **(D)** % fractional shortening, **(E)** cardiac output, **(F)** stroke volume, **(G)** LV end diastolic diameter (LVEDD) or **(H)** LV end systolic diameter (LVESD). *p < 0.05, **p < 0.01, ****p < 0.0001. Data shown as mean +/- SEM using one-way ANOVA with Holm-Šídák's multiple comparisons test with 5–10 mice/ group.

TUNEL Assay was performed to determine whether cardiac apoptosis was present 21 days after treatment with doxorubicin. We did not observe significant changes in apoptosis at day 21 after doxorubicin exposure between groups (Figure 3C). Fibrosis was found to be present in the heart at day 21 (Figure 4) and apoptosis is a process that primarily occurs prior to remodeling and fibrosis.

Vacuolation, a known effect of doxorubicin-induced cardiac damage in humans, was observed in male mice treated with doxorubicin (*p* < 0.0001, Figures 3D–H). *Trpc6*-deficiency significantly reduced cardiac vacuolation compared to wild-type controls following treatment with doxorubicin (*p* < 0.0001, Figures 3D–H), further demonstrating that *Trpc6* promotes cardiac damage following doxorubicin treatment.

Cardiac fibrosis is well known to cause cardiomyopathy/dilated cardiomyopathy that can be detected by echocardiography in conditions such as viral myocarditis (31, 32). Cardiac fibrosis was assessed at day 21. Wild-type mice treated with doxorubicin showed a significant increase in fibrosis in the heart (*p* = 0.010, Figure 4A) while *Trpc6*-deficiency significantly decreased fibrosis (*p* = 0.028, Figure 4A).

Cardiac function was measured in male mice at day 14 and 21 by echocardiography (Figure 4). No significant changes were observed for any group at day 14 (data not shown). At day 21, wild-type mice treated with doxorubicin showed a significant decrease in heart rate, (*p* = 0.029, Figure 4B), LVEF, (*p* = 0.042, Figure 4C), fractional shortening, (*p* = 0.037, Figure 4D), cardiac output, (*p* < 0.0001, Figure 4E) and stroke volume, (*p* = 0.0001, Figure 4F) compared to wild-type control males. *Trpc6*-deficiency significantly improved cardiac function compared to wild-type males treated with doxorubicin for heart rate *p* = 0.022, LVEF *p* = 0.048, fractional shortening *p* = 0.043, cardiac output *p* = 0.002, and stroke volume *p* = 0.048, respectively (Figures 4B–F). Measures of left ventricular end diastolic and left ventricular end systolic diameters (LVEDD, LVESD) used to determine cardiac dilatation showed that neither doxorubicin nor *Trpc6* deficiency led to dilated cardiomyopathy at this time point in males (Figures 4G,H). Thus, these data indicate that *Trpc6* promotes cardiac damage that leads to cardiomyopathy following doxorubicin treatment in males.

### In Male Mice, *Trpc6* Deficiency Improved *Trpc*-Related Gene Expression in the Heart Following Treatment With Doxorubicin

The TRPC family of proteins (TRPC1-7) function as both homo- and hetero-tetramers, and both *Trpc1* and *Trpc3* as well as *Trpc6* have been implicated in heart failure induced by pressure overload (23–25, 39). Another study reported that *Trpc6* is a positive regulator of calcineurin-NFAT signaling through the regulator of calcineurin (*Rcan1*) (40). Therefore, we sought to characterize the changes in cardiac gene expression of *Trpc6* in response to doxorubicin and *Trpc1, 3* and *Rcan1* in *Trpc6*-deficient mice after doxorubicin treatment.

**Figure 5:**
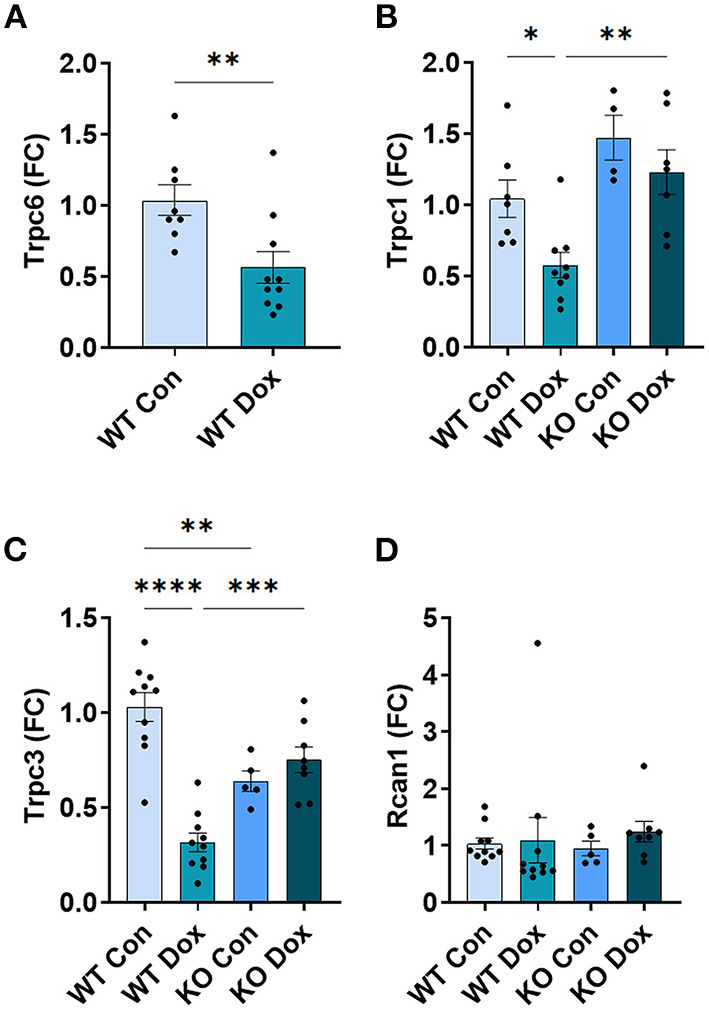
Other *Trpc* gene expression is not altered in *Trpc6*-deficient males after doxorubicin treatment. Male wild-type (WT) and *Trpc6*-deficient (KO) mice were treated with 100 mL saline (CON) or 4 mg/kg doxorubicin (DOX) on days 1, 3, 5, 7, 9, 11 for a cumulative dose of 24 mg/kg. Cardiac gene expression shown as a fold change (FC) relative to WT control for **(A)** *Trpc6*, **(B)** *Trpc1*, **(C)** *Trpc3* or **(D)** *Rcan1* at day 21. *p < 0.05, **p < 0.01, ***p < 0.001, ****p < 0.0001. **(A)** Data shown as mean ± SEM using 2 tailed Student's *t* test with 10 mice/ group. **(B–D)** Data shown as mean ± SEM using one-way ANOVA with Tukey's multiple comparisons test with 4–10 mice/ group.

In the hearts of male wild-type mice, we observed decreases in *Trpc6, Trpc1* and *Trpc3* gene expression in response to doxorubicin compared to saline controls, *p* = 0.0087, *p* = 0.032 and *p* < 0.0001, respectively (Figures 5A–C), but no significant change was observed in the expression of *Rcan1* (Figure 5D). In *Trpc6* deficient mice, the doxorubicin-induced changes in expression of *Trpc1* and *Trcp3* were reversed (Figures 5B,C). However, the expression of *Trpc3* in the hearts of *Trpc6* deficient control mice was significantly lower than that of wild-type control mice, (*p* = 0.004, Figure 5C), indicating that *Trpc6*-deficiency alters cardiac *Trpc3* expression regardless of doxorubicin treatment.

### Female Mice Are Less Susceptible to Doxorubicin-Induced Cardiac Damage, Cardiomyopathy and Death Compared to Males

**Figure 6:**
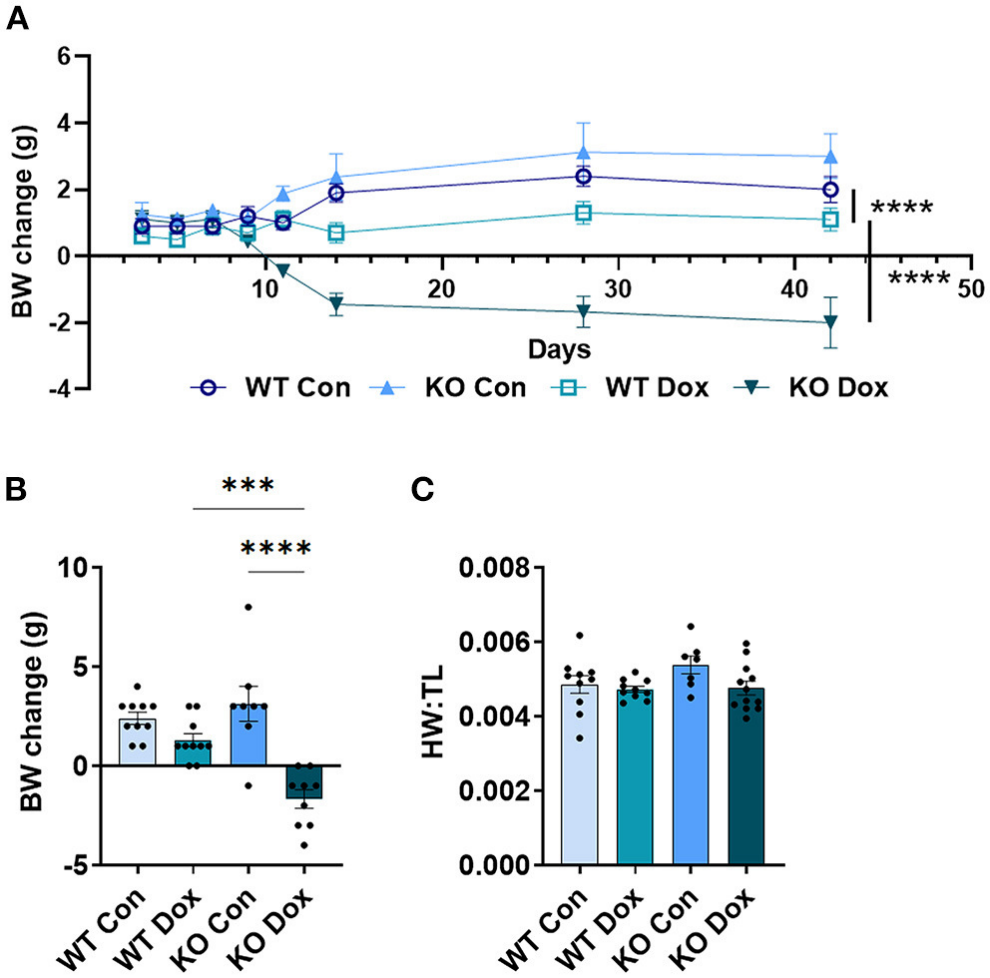
*Trpc6*-deficienct females treated with doxorubicin lose body weight, with no effect on heart weight. Female wild-type (WT) and *Trpc6-*deficient (KO) mice were treated with 100 mL saline (CON) or 4 mg/kg doxorubicin (DOX) on days 1, 3, 5, 7, 9, 11 for a cumulative dose of 24 mg/kg. **(A)** Change in body-weight (BW) from baseline in grams (g) over time. Data were analyzed by two-way ANOVA. **(B)** Change in body weight (BW) from baseline to day 21. **(C)** Ratio of heart weight to tibia length (HW:TL) at day 21. ***p < 0.001, ****p < 0.0001. Data shown as mean +/- SEM using one-way ANOVA with Tukey's multiple comparisons test with 7–12 mice/ group.

Given that women with breast cancer are commonly treated with doxorubicin and that our initial genetic studies identified *TRPC6* genetic variants as associated with a decline in LVEF in women with breast cancer (19), in this study we also assessed female mice treated with the same dose of doxorubicin as the dose given to males. In female wild-type mice, all wild-type and *Trpc6*-deficient mice survived treatment with doxorubicin (data not shown). In contrast to males, only *Trpc6*-deficient female mice treated with doxorubicin lost body weight over the duration of the experiment (Figure 6A). At day 21 (Figure 6B) wild-type female mice treated with doxorubicin maintained their weight, and no changes were observed in HW:TL in females for any group (Figure 6C).

**Figure 7:**
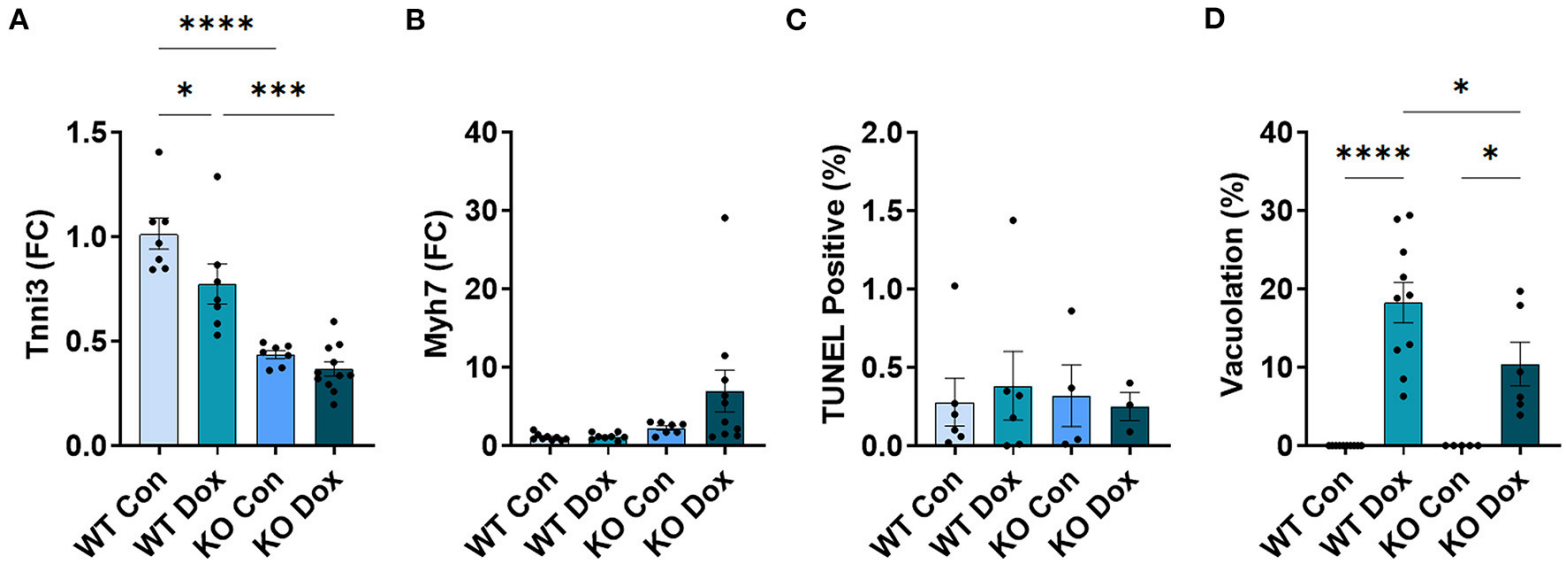
Trpc6-deficiency reduces cardiac damage in females. Female wild-type (WT) and *Trpc6*-deficient (KO) mice were treated with 100 mL saline (CON) or 4 mg/kg doxorubicin (DOX) on days 1, 3, 5, 7, 9, 11 for a cumulative dose of 24 mg/kg. **(A)** Cardiac troponin (*Tnni3*) or **(B)** myosin heavy chain 7 (*Myh7*) gene expression shown as a fold change (FC) relative to WT control. **(C)** TUNEL Positive (%) **(D)** Percent vacuolation. *p < 0.05, ***p < 0.001, ****p < 0.0001. Data shown as mean +/- SEM using one-way ANOVA with Tukey's multiple comparisons test with 4–12 mice/ group.

**Figure 8:**
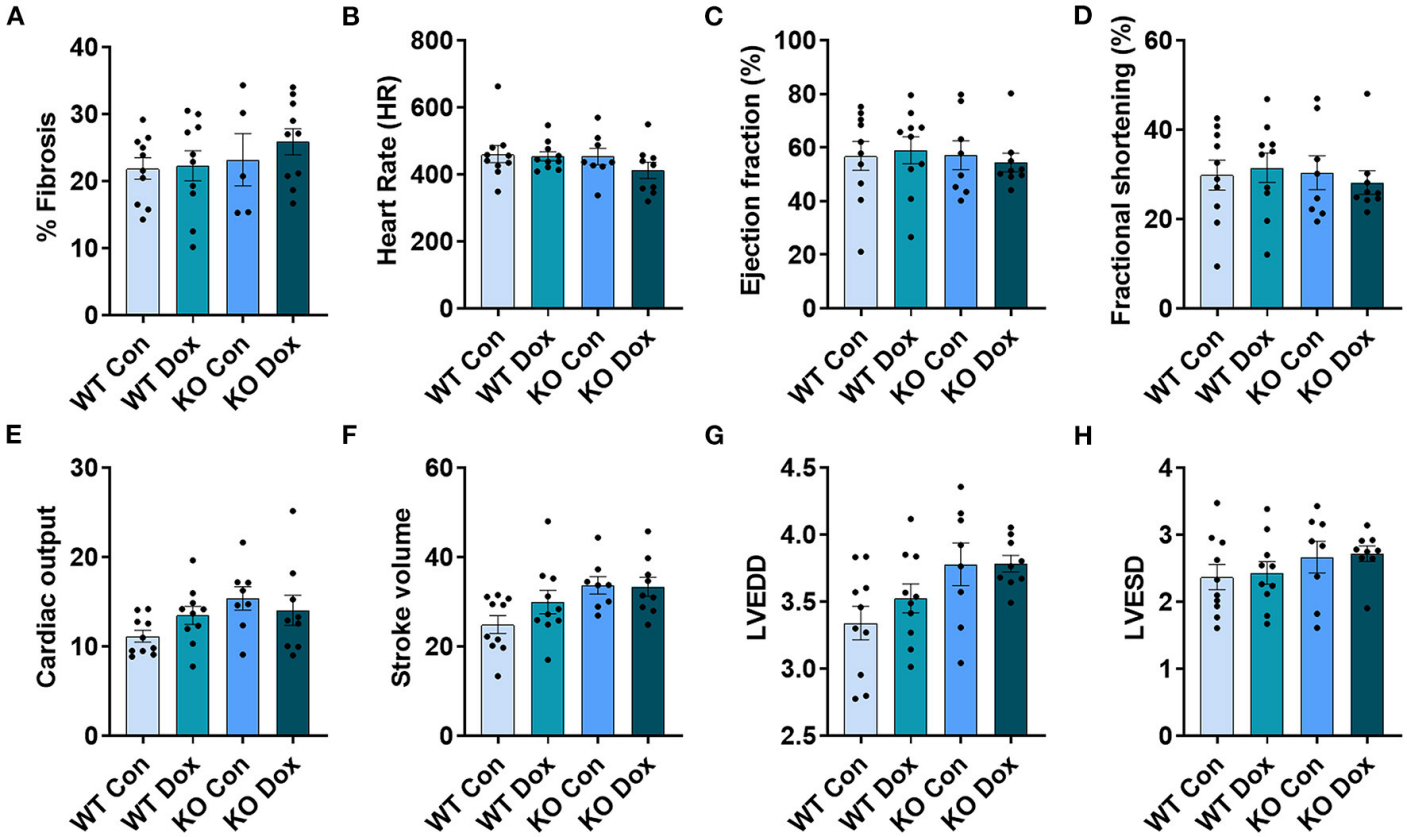
In female mice, cardiac function measured by echocardiography was not impacted by doxorubicin or Trpc6 deficiency. Female wild-type (WT) and *Trpc6*-deficient (KO) mice were treated with 100 mL saline (CON) or 4 mg/kg doxorubicin (DOX) on days 1, 3, 5, 7, 9, 11 for a cumulative dose of 24 mg/kg. Trichrome blue staining was performed at day 21 to assess **(A)** % fibrosis. Echocardiogram was performed at day 21 to assess **(B)** heart rate (HR), **(C)** % left ventricular (LV) ejection fraction, **(D)** % fractional shortening, **(E)** cardiac output, **(F)** stroke volume, **(G)** LV end diastolic diameter (LVEDD) or **(H)** LV end systolic diameter (LVESD). Data shown as mean ± SEM using one-way ANOVA with Tukey's multiple comparisons with 5–10 mice/ group.

As observed in male mice, *Tnni3* cardiac gene expression was significantly reduced in wild-type females treated with doxorubicin (*p* = 0.050, Figure 7A), but unlike males, gene expression of *Myh7* in wild-type females was not significantly altered by doxorubicin, (*p* > 0.999, Figure 7B). Similar to males, female mice developed vacuolation following treatment with doxorubicin, (*p* < 0.0001, Figure 7D) that was less severe than males (mean vacuolation in wild-type females treated with doxorubicin = 18.24% vs. 74.44% in males) (Figure 7D). And as with males, *Trpc6*-deficiency significantly reduced vacuolation in females treated with doxorubicin (*p* = 0.049, Figure 7D). Finally, we did not observe any significant change in cardiac fibrosis or echocardiographic parameters in female mice at day 21 in response to doxorubicin or *Trpc6*-deficiency (Figure 8). Thus, cardiac damage caused by doxorubicin was far less in females and did not lead to cardiomyopathy at day 21.

### Pleiotropic Effects of Trpc6 Deficiency in Female Mice Following Doxorubicin Treatment

Although female wild-type and *Trpc6*-deficient mice were less susceptible to doxorubicin-induced cardiac damage and cardiomyopathy, we did observe other significant effects of *Trpc6* deficiency in female mice compared to males.

In contrast to males (Figure 2A), female wild-type mice treated with doxorubicin did not lose weight (Figure 6A). The reason for this is not clear. Rather than wild-type mice being worse in males, *Trpc6*-deficient females treated with doxorubicin had a greater loss in body weight over time and at day 21 compared to wild-type mice treated with doxorubicin (*p* < 0.0001, Figures 6A,B). Although doxorubicin significantly decreased HW:TL (caused heart damage) in males (Figure 2C), there was no change in heart weight (no cardiac damage) in females (Figure 6C).

In contrast to males (Figure 3A), *Tnni3* cardiac gene expression was significantly lower in *Trpc6*-deficient compared to wild-type saline control females (*p* < 0.0001, Figure 7A). In contrast to males, *Tnni3* gene expression was significantly decreased in control and doxorubicin treated *Trpc6*-deficient females (Figure 7A), suggesting that *Trpc6* deficiency altered *Tnni3* levels in females. Pleiotropic effects of *Trpc6* deficiency in response to doxorubicin were also observed for *Myh7* gene expression in the hearts of female (Figure 7B) vs. male (Figure 3B) mice. In female mice, *Myh7* levels remained low in all groups except for *Trpc6*-deficient mice treated with doxorubicin, where there was a significant increase relative to wild-type controls, (*p* = 0.049, Figure 7B).

**Figure 9:**
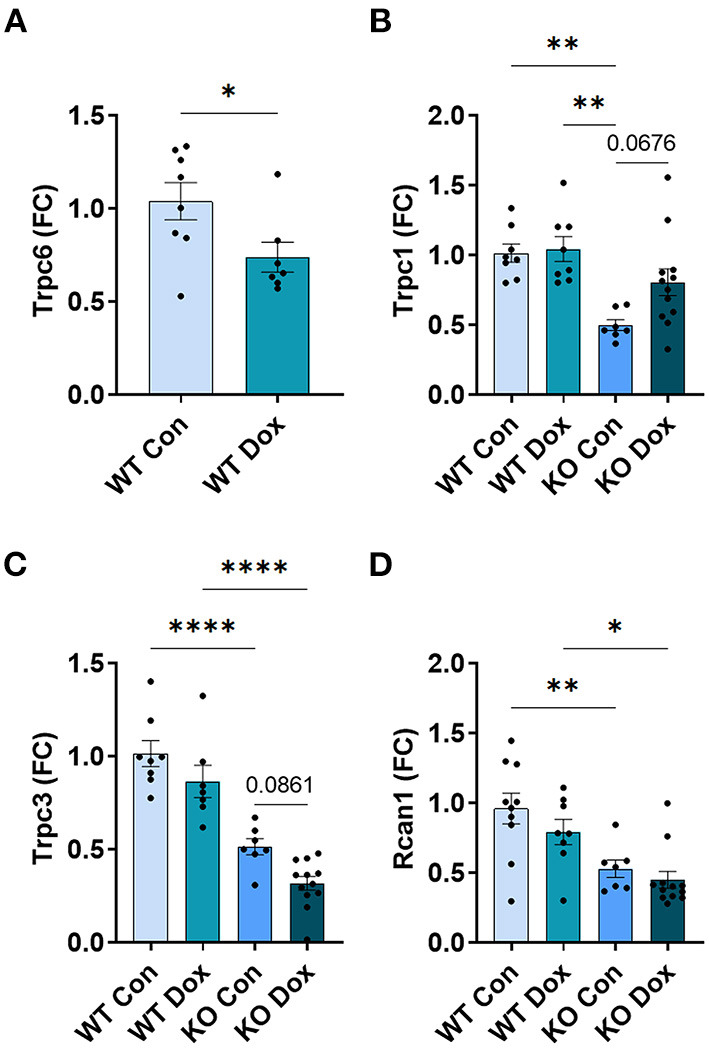
*Trpc6* expression is reduced by doxorubicin in female wild-type mice while *Trpc1, Trpc3* and *Rcan1* expression is reduced by *Trpc6* deficiency. Female wild-type (WT) and *Trpc6*-deficient (KO) mice were treated with 100 mL saline (CON) or 4 mg/kg doxorubicin (DOX) on days 1, 3, 5, 7, 9, 11 for a cumulative dose of 24 mg/kg. Cardiac gene expression shown as a fold change (FC) relative to WT control **(A)** *Trpc6*, **(B)** *Trpc1*, **(C)** *Trpc3* and **(D)** *Rcan1* at day 21. *p < 0.05, **p < 0.01, ****p < 0.0001. Data shown as mean ± SEM using one-way ANOVA with Tukey's multiple comparisons with 7–12 mice/ group.

**Figure 10:**
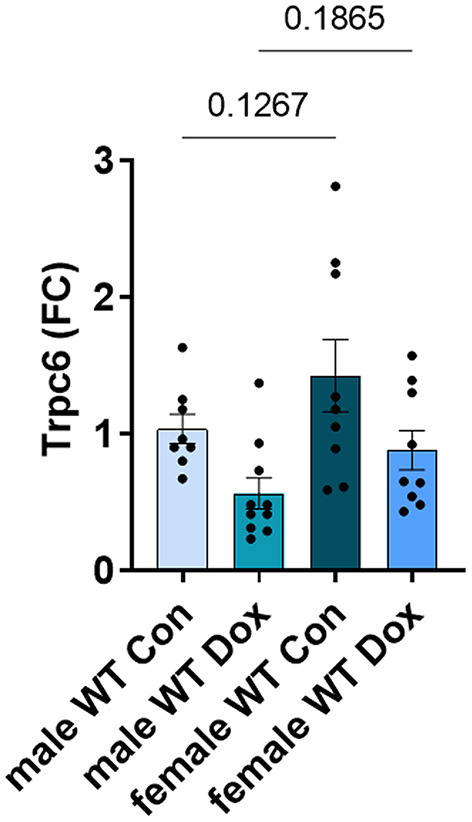
*Trpc6* expression was not significantly different between male and female wild-type control mice. Female and male wild-type (WT) and *Trpc6*-deficient (KO) mice were treated with 100 mL saline (CON) or 4 mg/kg doxorubicin (DOX) on days 1, 3, 5, 7, 9, 11 for a cumulative dose of 24 mg/kg. Cardiac gene expression of *Trpc6* shown as a fold change (FC) relative to male WT control at day 21. Data shown as mean ± SEM using one-way ANOVA with Fisher's LSD multiple comparisons test with 8–10 mice/ group.

In the hearts of female wild-type mice, doxorubicin induced a significant reduction in *Trpc6* gene expression compared to wildtype controls (*p* = 0.039, Figure 9A) similar to the decrease observed in male mice (Figure 5A), but did not induce changes in *Trpc1, Trpc3* or *Rcan1* in wild-type mice (Figures 9B–D). A direct comparison of Trpc6 expression levels in the heart of male and female wild-type mice revealed that there were no significant differences in its expression before or after treatment with doxorubicin by sex (Figure 10). Interestingly, female *Trpc6*-deficient mice treated with saline had significantly lower expression of *Trpc1*, (*p* = 0.002, Figure 9B), *Trpc3* (*p* < 0.0001, Figure 9C) and *Rcan1* (*p* = 0.009, Figure 9D) than wild-type control mice. Thus overall, *Trpc6* appears to increase cardiac damage in response to doxorubicin in females but not severely enough to lead to cardiomyopathy at the dose used in these experiments.

## Discsussion

In breast cancer patients, genetic variants at *TRPC6* have been associated with doxorubicin-induced cardiomyopathy and congestive heart failure (19, 20). *In vitro* and *in vivo, Trpc6* inhibition with the peptide GsMTx4 reduced doxorubicin-induced cardiotoxicity and cardiomyopathy in male mice (20). However, GsMTx4 also inhibits Piezo 1 and Piezo 2 (41) and TRPC1 (42) such that its cardioprotective effects could be mediated through mechanosensitive ion channels other than Trpc6 or the combination of multiple ion channels.

In this study, we specifically tested the role of *Trpc6* in doxorubicin-induced cardiotoxicity and cardiomyopathy using male and female *Trpc6* whole body knockout mice. In male mice in this study, we found that *Trpc6* deficiency improved doxorubicin-induced cardiac damage (vacuolation, fibrosis, *Tnni3* and *Myh7*) and cardiomyopathy indicating that Trpc6 promotes cardiac damage associated with doxorubicin therapy. To our knowledge, our study is the first to examine the effect of *Trpc6* in doxorubicin-induced cardiomyopathy. Seo et al. (24) found that *Trpc6* deficiency in male mice had no significant effect on hypertrophy induced using a pressure overload model by transverse aortic constriction (TAC). Improvement in hypertrophy following TAC required combined *Trpc3* and *Trpc6* deficiency (24). There are several reasons for differences between our study and Seo *et al*. Firstly, the genes and mechanisms involved in doxorubicin-induced heart failure are different than those in pressure overload models, and mice treated with doxorubicin in this study did not show signs of hypertrophic cardiomyopathy according to measurement of heart weight to tibia length. Secondly, some mouse strains are more sensitive to heart failure than others (both our study and Seo *et al*. used *Trpc6* whole body knockout mice (26), but in the Seo study Trpc6 KO mice were backcrossed onto a C57BL/6J background and the mice in this study were on a B6.129 background). Thirdly, it is possible that combined *Trpc3* and *Trpc6* deficiency could improve cardioprotection even further in mice treated with doxorubicin.

In the KO male and female mice in this study, we also observed that *Trpc3* expression was significantly lower in *Trpc6* KO controls relative to wild-type controls raising the possibility that at least some of the protective effect of *Trpc6* deficiency could be mediated by decreased expression of *Trpc3*. Indeed, the work of others in pressure overload models of heart failure demonstrated that Pyr3- specific inhibition of *Trpc3* attenuated pressure overload-induced heart failure in male mice (43), and the same group demonstrated that inhibition of the *Trcp3-Nox2* complex suppressed doxorubicin-induced myocardial atrophy (44). However, an independent group demonstrated that both deletion and inhibition of *Trpc6* reduced pressure overload-induced fibrosis, but did not reduce pressure overload-induced cardiac dysfunction or ROS production (45), and a recent *in vitro* study found that doxorubicin-induced cell death was independent of TRPC6 channel up-regulation but involved mitochondrial activation of ROS (46). Taken together, our data and that of others suggest that cardioprotection through Trpc6 deficiency may be mediated by reduction of fibrosis (our previous work showed that doxorubicin-induced fibrosis was reduced in mice that were pre-treated with the Trpc6 inhibitor GsMTx4, which does not inhibit Trpc3) as well as by reduction in *Trpc3* expression.

We are also the first study to our knowledge to examine whether sex differences exist in the effect of *Trpc6* on cardiomyopathy following doxorubicin therapy. We found that female B6.129 wild-type and *Trpc6*-deficient mice were far less susceptible to doxorubicin-induced cardiac damage and cardiomyopathy than males. This is consistent with the known sex differences in cardiomyopathy and heart failure where male mice and men develop worse cardiac function than females (47, 48). In animal models of heart disease, estrogen has been found to improve cardiac function in females (30, 49). Specifically, estrogen has been found to prevent cardiac hypertrophy by reducing calcineurin activity (50). Age also influences sex differences, with cardiovascular disease increasing in women after menopause and with increasing age (after 70 years of age) (51, 52). Additionally, older women (age >65 years, which accounts for 50% of breast cancer cases) are at higher risk of chemotherapy-related heart failure compared to younger women (53, 54) and the mice used in this study were 8-10 week old young adults. Furthermore, other female rodent models have shown that ovariectomy is necessary for the development of heart failure (55). Future studies should examine whether ovariectomized female mice treated with doxorubicin develop more severe cardiac damage and cardiomyopathy.

In this study, we found that doxorubicin reduced *Trpc6* gene expression in both male and female wild-type mice, suggesting a homeostatic response to buffer Trpc6-induced damage. We observed that *Trpc1* and *Trpc3* expression were also significantly reduced in male wild-type mice in response to doxorubicin, but this did not occur in females. As female mice were much less susceptible to doxorubicin-induced cardiac damage, they may have greater ability to buffer Trpc6-induced changes in calcium levels. Sex hormones have been found to influence calcium channels (56, 57), and 17b-estradiol to upregulate canonical transient receptor potential channels (TRPC) in particular (58). This could explain the reduction in cardiac *Trpc1, Trpc3* and *Rcan1* gene expression in *Trpc6*-deficient saline control and doxorubicin-treated female mice that was not observed in males. *Rcan1* is involved in both development and maintenance of the cardiovascular system, and reduction in *Rcan1* prevents pathological cardiac remodeling (59, 60). In this study, *Rcan1* expression was unchanged in male mice in response to doxorubicin or *Trpc6*-deficiency, but in *Trpc6*-deficient female mice, *Rcan1* expression was significantly reduced in females and the pattern of expression closely followed that of *Trpc3* in each group of mice by treatment and *Trpc6* status.

The observed sex differences in *Trpc* channel expression in response to doxorubicin and *Trpc6*-deficiency suggest that estrogen is not only cardioprotective, but perhaps the mechanism of estrogen-related cardioprotection is mediated through TRPC-related calcium signaling in the heart. Regulation of TRPC gene expression by estrogen was first reported in 1997 (61) and both E_2_ and the G-protein estrogen receptor (GPER) act to moderate calcium-activities in the cardiovascular system by lowering the peaks and raising the troughs, thus refining calcium levels to a more narrow and sustained operating range [reviewed in (62)]. Taken together, these data suggest that TRPC6 inhibition may serve as a potential cardioprotective therapy for male and post-menopausal female cancer patients that require doxorubicin.

Additionally, other work from our group identified genetic variants that were associated with both chemotherapy-induced heart failure and a decline in LVEF, and the same variants were also associated with increased TRPC6 expression in the heart, and in one case we identified a TRPC6 gain-of-function variant in a 32 year old women with breast cancer who developed heart failure following doxorubicin and trastuzumab treatment (20) and (63) suggesting that TRPC6 inhibition may be particularly appropriate as a cardioprotection strategy for men and women who carry *TRPC6* risk variants.

Our use of both female and male mice also demonstrated a significant interaction in female mice between doxorubicin and *Trpc6* that may be clinically relevant to the management of patients receiving anthracyclines. The most striking sex difference was the loss of body-weight over time, in which male wild-type mice treated with doxorubicin lost a significant amount of body-weight whereas female wild-type mice were almost completely unaffected. In male mice, *Trpc6*-deficiency did not prevent doxorubicin-induced weight loss, but in female mice *Trpc6*-deficiency had a dramatic effect on weight gain in mice that received only saline control and a dramatic effect on weight loss in mice that were treated with doxorubicin, demonstrating genetic pleiotropy of *Trpc6* deficiency in response to doxorubicin, specifically in female mice. These changes in body-weight appeared independent to changes in heart weight or cardiac function. However, we note that TRPC channels are often overexpressed in tumors, are a hallmark of metastasis (64) and *in vitro*, TRPC channel knock down or inhibition reduces tumor cell growth and migration (65, 66). Therefore, our observations of the pleiotropic effects of *Trpc6*-deficiency in female mice in relation to doxorubicin treatment may be of clinical relevance to the efficacy of doxorubicin in breast cancer patients. For example, would *Trpc6* inhibition make breast tumors more sensitive to doxorubicin or prevent resistance to doxorubicin?

In summary, we demonstrated that *Trpc6* deficiency alone is sufficient to protect male mice from doxorubicin-induced cardiac damage and decline in cardiac function, suggesting that TRPC6 may be a valuable therapeutic target for cancer patients who require doxorubicin. We further showed that wild-type female mice are less susceptible to doxorubicin-induced damage, with low levels of cardiomyocyte vacuolation and no progression to cardiomyopathy at doses used in this study. Finally, we demonstrated significant sex differences in the gene expression of *Trpc1, Trpc3* and *Rcan1* in wild-type and *Trpc6*-deficient mice that may be due to the reciprocal relationship between estrogen and TRPC activity, which is of relevance to cardioprotection strategies for women with breast cancer.

## Conclusions

*Trpc6* induces cardiac damage and cardiomyopathy following treatment with doxorubicin in male mice and may be a therapeutic target for cardioprotection in patients. Female mice are less susceptible to cardiotoxicity but showed cardioprotection in *Trpc6*-deficient mice.

## Data Availability Statement

The original contributions presented in the study are included in the article/supplementary materials, further inquiries can be directed to the corresponding authors.

## Ethics Statement

The animal study was reviewed and approved by Mayo Clinic, American Association for Laboratory Animal Science.

## Author Contributions

NN, KB, and DF designed the animal experiments. KB, DD, EW, AH, AM-L, AM, JS, JM, LD, GS, ZA, LM, SK, MM, PG, AK, DB, and DF performed animal experiments and animal maintenance. NN, KB, DD, EW, MM, AM-L, and AM performed doxorubicin dosing, weight tracking and collected survival data. KB, DD, EW, and AH performed echocardiography. KB, DD, EW, JS, and CL analyzed echocardiography data. KB and SK performed tibia length measurements. KB, LM, and DF performed histological analysis. NN, KB, AH, JM, LD, GS, ZA, BN, RW, and DF performed RT-PCR experiments. DD, LM, and SK performed TUNEL Assay. NN, KB, and DF interpreted the data and wrote the manuscript. All authors critically revised the manuscript.

## Funding

This work was funded by a Mayo Clinic Cardiovascular Team Science Award (NN, DF and CL), National Institutes of Health (NIH) grant TL1 TR002380 (DD) and National Institute of Allergy and Infectious Disease (NIAID) grants R21 AI145356, R21 AI152318, R21 AI154927 and American Heart Association grant 20TPA35490415 (DF).

## Conflict of Interest

The authors declare that the research was conducted in the absence of any commercial or financial relationships that could be construed as a potential conflict of interest.

## Publisher's Note

All claims expressed in this article are solely those of the authors and do not necessarily represent those of their affiliated organizations, or those of the publisher, the editors and the reviewers. Any product that may be evaluated in this article, or claim that may be made by its manufacturer, is not guaranteed or endorsed by the publisher.

## Abbreviations

CHF: congestive heart failure
ROS: reactive oxygen species
LVEF: left ventricular ejection fraction
TRPC6: transient receptor potential cation channel subunit 6
CHF: congestive heart failure
WT: wild-type
KO: knock-out
ip: intraperitoneally
qPCR: quantitative reverse transcriptase-mediated real-time PCR
(qRT)-PCR: quantitative real time
pi: post inoculation.

## References

[1] Drafts BC Twomley KM D'Agostino R Jr. Lawrence J Avis N Ellis LR. Low to moderate dose anthracycline-based chemotherapy is associated with early noninvasive imaging evidence of subclinical cardiovascular disease. JACC Cardiovasc Imaging. (2013) 6:877–85. 10.1016/j.jcmg.2012.11.017 23643285 PMC3745801

[2] Lipshultz SE Adams MJ. Cardiotoxicity after childhood cancer: beginning with the end in mind. J Clin Oncol. (2010) 28:1276–81. 10.1200/JCO.2009.26.5751 20142585

[3] Lipshultz SE Lipsitz SR Sallan SE Dalton VM Mone SM Gelber RD. Chronic progressive cardiac dysfunction years after doxorubicin therapy for childhood acute lymphoblastic leukemia. J Clin Oncol. (2005) 23:2629–36. 10.1200/JCO.2005.12.121 15837978

[4] Plana JC. The red devil revisited. Jacc-Cardiovasc Imag. (2013) 6:886–8. 10.1016/j.jcmg.2013.04.009 23948379

[5] Swain SM Whaley FS Ewer MS. Congestive heart failure in patients treated with doxorubicin: a retrospective analysis of three trials. Cancer. (2003) 97:2869–79. 10.1002/cncr.11407 12767102

[6] Von Hoff DD Layard MW Basa P Davis HL Jr Von Hoff AL Rozencweig M. Risk factors for doxorubicin-induced congestive heart failure. Ann Intern Med. (1979) 91:710–7. 10.7326/0003-4819-91-5-710 496103

[7] Simunek T Sterba M Popelova O Adamcova M Hrdina R Gersl V. Anthracycline-induced cardiotoxicity: overview of studies examining the roles of oxidative stress and free cellular iron. Pharmacol Rep. (2009) 61:154–71. 10.1016/S1734-1140(09)70018-0 19307704

[8] Zhang S Liu X Bawa-Khalfe T Lu LS Lyu YL Liu LF. Identification of the molecular basis of doxorubicin-induced cardiotoxicity. Nat Med. (2012) 18:1639–42. 10.1038/nm.2919 23104132

[9] Zhou S Starkov A Froberg MK Leino RL Wallace KB. Cumulative and irreversible cardiac mitochondrial dysfunction induced by doxorubicin. Cancer Res. (2001) 61:771–7. 11212281

[10] Lebrecht D Kirschner J Geist A Haberstroh J Walker UA. Respiratory chain deficiency precedes the disrupted calcium homeostasis in chronic doxorubicin cardiomyopathy. Cardiovasc Pathol. (2010) 19:e167–74. 10.1016/j.carpath.2009.06.006 19747854

[11] Zhou S Heller LJ Wallace KB. Interference with calcium-dependent mitochondrial bioenergetics in cardiac myocytes isolated from doxorubicin-treated rats. Toxicol Appl Pharmacol. (2001) 175:60–7. 10.1006/taap.2001.9230 11509027

[12] Solem LE Henry TR Wallace KB. Disruption of mitochondrial calcium homeostasis following chronic doxorubicin administration. Toxicol Appl Pharmacol. (1994) 129:214–22. 10.1006/taap.1994.1246 7527602

[13] Zhao L Zhang B. Doxorubicin induces cardiotoxicity through upregulation of death receptors mediated apoptosis in cardiomyocytes. Sci Rep. (2017) 7:44735. 10.1038/srep44735 28300219 PMC5353581

[14] Bien S Riad A Ritter CA Gratz M Olshausen F Westermann D. The endothelin receptor blocker bosentan inhibits doxorubicin-induced cardiomyopathy. Cancer Res. (2007) 67:10428–35. 10.1158/0008-5472.CAN-07-1344 17974986

[15] Armenian SH Lacchetti C Barac A Carver J Constine LS Denduluri N. Prevention and monitoring of cardiac dysfunction in survivors of adult cancers: american society of clinical oncology clinical practice guideline. J Clin Oncol. (2017) 35:893–911. 10.1200/JCO.2016.70.5400 27918725

[16] Deng S Yan T Jendrny C Nemecek A Vincetic M Godtel-Armbrust U. Dexrazoxane may prevent doxorubicin-induced DNA damage via depleting both topoisomerase II isoforms. BMC Cancer. (2014) 14:842. 10.1186/1471-2407-14-842 25406834 PMC4242484

[17] Hasinoff BB Patel D Wu X. The role of topoisomerase IIbeta in the mechanisms of action of the doxorubicin cardioprotective agent dexrazoxane. Cardiovasc Toxicol. (2019). 20:312–20. 10.1007/s12012-019-09554-5 31773441

[18] Swain SM Whaley FS Gerber MC Weisberg S York M Spicer D. Cardioprotection with dexrazoxane for doxorubicin-containing therapy in advanced breast cancer. J Clin Oncol. (1997) 15:1318–32. 10.1200/JCO.1997.15.4.1318 9193323

[19] Serie DJ Crook JE Necela BM Dockter TJ Wang X Asmann YW. Genome-wide association study of cardiotoxicity in the NCCTG N9831 (Alliance) adjuvant trastuzumab trial. Pharmacogenet Genomics. (2017) 10:378–85. 10.1097/FPC.0000000000000302 28763429 PMC5581215

[20] Norton N Crook JE Wang L Olson JE Kachergus JM Serie DJ. Association of genetic variants at TRPC6 with chemotherapy-related heart failure. Front Cardiovasc Med. (2020) 7:142. 10.3389/fcvm.2020.00142 32903434 PMC7438395

[21] Beech DJ. Characteristics of transient receptor potential canonical calcium-permeable channels and their relevance to vascular physiology and disease. Circ J. (2013) 77:570–9. 10.1253/circj.CJ-13-0154 23412755

[22] Polat OK Uno M Maruyama T Tran HN Imamura K Wong CF. Contribution of Coiled-Coil Assembly to Ca(2+)/Calmodulin-Dependent Inactivation of TRPC6 Channel and its Impacts on FSGS-Associated Phenotypes. J Am Soc Nephrol. (2019) 30:1587–603. 10.1681/ASN.2018070756 31266820 PMC6727271

[23] Numaga-Tomita T Kitajima N Kuroda T Nishimura A Miyano K Yasuda S. TRPC3-GEF-H1 axis mediates pressure overload-induced cardiac fibrosis. Sci Rep. (2016) 6:39383. 10.1038/srep39383 27991560 PMC5171702

[24] Seo K Rainer PP Shalkey Hahn V Lee DI Jo SH Andersen A. Combined TRPC3 and TRPC6 blockade by selective small-molecule or genetic deletion inhibits pathological cardiac hypertrophy. Proc Natl Acad Sci U S A. (2014) 111:1551–6. 10.1073/pnas.1308963111 24453217 PMC3910575

[25] Seth M Zhang ZS Mao L Graham V Burch J Stiber J. TRPC1 channels are critical for hypertrophic signaling in the heart. Circ Res. (2009) 105:1023–30. 10.1161/CIRCRESAHA.109.206581 19797170 PMC2881555

[26] Dietrich A Mederos YSM Gollasch M Gross V Storch U Dubrovska G. Increased vascular smooth muscle contractility in TRPC6-/- mice. Mol Cell Biol. (2005) 25:6980–9. 10.1128/MCB.25.16.6980-6989.2005 16055711 PMC1190236

[27] Abston ED Barin JG Cihakova D Bucek A Coronado MJ Brandt JE. IL-33 independently induces eosinophilic pericarditis and cardiac dilation: ST2 improves cardiac function. Circ Heart Fail. (2012) 5:366–75. 10.1161/CIRCHEARTFAILURE.111.963769 22454393 PMC3874395

[28] Abston ED Coronado MJ Bucek A Bedja D Shin J Kim JB. Th2 regulation of viral myocarditis in mice: different roles for TLR3 versus TRIF in progression to chronic disease. Clin Dev Immunol. (2012) 2012:129486. 10.1155/2012/129486 22013485 PMC3195533

[29] Bruno KA Mathews JE Yang AL Frisancho JA Scott AJ Greyner HD. BPA alters estrogen receptor expression in the heart after viral infection activating cardiac mast cells and T cells leading to perimyocarditis and fibrosis. Front Endocrinol (Lausanne). (2019) 10:598. 10.3389/fendo.2019.00598 31551929 PMC6737078

[30] Coronado MJ Bruno KA Blauwet LA Tschope C Cunningham MW Pankuweit S. Elevated Sera sST 2 Is Associated With Heart Failure in Men </=50 years old with myocarditis. J Am Heart Assoc. (2019) 8:e008968. 10.1161/JAHA.118.008968 30638108 PMC6497352

[31] Fairweather D Frisancho-Kiss S Njoku DB Nyland JF Kaya Z Yusung SA. Complement receptor 1 and 2 deficiency increases coxsackievirus B3-induced myocarditis, dilated cardiomyopathy, and heart failure by increasing macrophages, IL-1beta, and immune complex deposition in the heart. J Immunol. (2006) 176:3516–24. 10.4049/jimmunol.176.6.3516 16517720

[32] Fairweather D Frisancho-Kiss S Yusung SA Barrett MA Davis SE Gatewood SJ. Interferon-gamma protects against chronic viral myocarditis by reducing mast cell degranulation, fibrosis, and the profibrotic cytokines transforming growth factor-beta 1, interleukin-1 beta, and interleukin-4 in the heart. Am J Pathol. (2004) 165:1883–94. 10.1016/S0002-9440(10)63241-5 15579433 PMC1618717

[33] Radonic A Thulke S Mackay IM Landt O Siegert W Nitsche A. Guideline to reference gene selection for quantitative real-time PCR. Biochem Biophys Res Commun. (2004) 313:856–62. 10.1016/j.bbrc.2003.11.177 14706621

[34] Livak KJ Schmittgen TD. Analysis of relative gene expression data using real-time quantitative PCR and the 2(-Delta Delta C(T)) Method. Methods. (2001) 25:402–8. 10.1006/meth.2001.1262 11846609

[35] Miyata S Minobe W Bristow MR Leinwand LA. Myosin heavy chain isoform expression in the failing and nonfailing human heart. Circ Res. (2000) 86:386–90. 10.1161/01.RES.86.4.386 10700442

[36] Nakao K Minobe W Roden R Bristow MR Leinwand LA. Myosin heavy chain gene expression in human heart failure. J Clin Invest. (1997) 100:2362–70. 10.1172/JCI119776 9410916 PMC508434

[37] Boluyt MO O'Neill L Meredith AL Bing OH Brooks WW Conrad CH. Alterations in cardiac gene expression during the transition from stable hypertrophy to heart failure. Marked upregulation of genes encoding extracellular matrix components. Circ Res. (1994) 75:23–32. 10.1161/01.RES.75.1.23 8013079

[38] Mercadier JJ Lompre AM Wisnewsky C Samuel JL Bercovici J Swynghedauw B. Myosin isoenzyme changes in several models of rat cardiac hypertrophy. Circ Res. (1981) 49:525–32. 10.1161/01.RES.49.2.525 6454511

[39] Kitajima N Numaga-Tomita T Watanabe M Kuroda T Nishimura A Miyano K. TRPC3 positively regulates reactive oxygen species driving maladaptive cardiac remodeling. Sci Rep. (2016) 6:37001. 10.1038/srep37001 27833156 PMC5105134

[40] Kuwahara K Wang Y McAnally J Richardson JA Bassel-Duby R Hill JA. TRPC6 fulfills a calcineurin signaling circuit during pathologic cardiac remodeling. J Clin Invest. (2006) 116:3114–26. 10.1172/JCI27702 17099778 PMC1635163

[41] Suchyna TM. Piezo channels and GsMTx4: Two milestones in our understanding of excitatory mechanosensitive channels and their role in pathology. Prog Biophys Mol Biol. (2017) 130(Pt B):244–53. 10.1016/j.pbiomolbio.2017.07.011 28778608 PMC5716857

[42] Spassova MA Hewavitharana T Xu W Soboloff J Gill DL. A common mechanism underlies stretch activation and receptor activation of TRPC6 channels. Proc Natl Acad Sci U S A. (2006) 103:16586–91. 10.1073/pnas.0606894103 17056714 PMC1637625

[43] Kitajima N Watanabe K Morimoto S Sato Y Kiyonaka S Hoshijima M. TRPC3-mediated Ca2+ influx contributes to Rac1-mediated production of reactive oxygen species in MLP-deficient mouse hearts. Biochem Biophys Res Commun. (2011) 409:108–13. 10.1016/j.bbrc.2011.04.124 21565173

[44] Shimauchi T Numaga-Tomita T Ito T Nishimura A Matsukane R Oda S. TRPC3-Nox2 complex mediates doxorubicin-induced myocardial atrophy. JCI Insight. (2017) 2:e93358. 10.1172/jci.insight.93358 28768915 PMC5543921

[45] Oda S Numaga-Tomita T Kitajima N Toyama T Harada E Shimauchi T. TRPC6 counteracts TRPC3-Nox2 protein complex leading to attenuation of hyperglycemia-induced heart failure in mice. Sci Rep. (2017) 7:7511. 10.1038/s41598-017-07903-4 28790356 PMC5548754

[46] Matthews AT Soni H Robinson-Freeman KE John TA Buddington RK Adebiyi A. Doxorubicin-induced fetal mesangial cell death occurs independently of TRPC6 channel upregulation but involves mitochondrial generation of reactive oxygen species. Int J Mol Sci. (2021) 22:7589. 10.3390/ijms22147589 34299212 PMC8305841

[47] da Silva JS Montagnoli TL Rocha BS Tacco M Marinho SCP Zapata-Sudo G. Estrogen receptors: therapeutic perspectives for the treatment of cardiac dysfunction after myocardial infarction. Int J Mol Sci. (2021) 22:525. 10.3390/ijms22020525 33430254 PMC7825655

[48] Fairweather D Cooper LT Jr Blauwet LA. Sex and gender differences in myocarditis and dilated cardiomyopathy. Curr Probl Cardiol. (2013) 38:7–46. 10.1016/j.cpcardiol.2012.07.003 23158412 PMC4136454

[49] Firth JM Yang HY Francis AJ Islam N MacLeod KT. The effect of estrogen on intracellular Ca(2+) and Na(+) regulation in heart failure. JACC Basic Transl Sci. (2020) 5:901–12. 10.1016/j.jacbts.2020.06.013 33015413 PMC7524784

[50] Pedram A Razandi M Aitkenhead M Levin ER. Estrogen inhibits cardiomyocyte hypertrophy in vitro. Antagonism of calcineurin-related hypertrophy through induction of MCIP1. J Biol Chem. (2005) 280:26339–48. 10.1074/jbc.M414409200 15899894 PMC1249515

[51] Sabbatini AR Kararigas G. Estrogen-related mechanisms in sex differences of hypertension and target organ damage. Biol Sex Differ. (2020) 11:31. 10.1186/s13293-020-00306-7 32487164 PMC7268741

[52] Virani SS Alonso A Aparicio HJ Benjamin EJ Bittencourt MS Callaway CW. Heart disease and stroke statistics-2021 update: a report from the american heart association. Circulation. (2021) 143:e254–743. 10.1161/CIR.0000000000000950 33501848 PMC13036842

[53] Advani PP Ballman KV Dockter TJ Colon-Otero G Perez EA. Long-term cardiac safety analysis of NCCTG N9831 (Alliance) adjuvant trastuzumab trial. J Clin Oncol. (2016) 34:581–7. 10.1200/JCO.2015.61.8413 26392097 PMC4980566

[54] Romond EH Jeong JH Rastogi P Swain SM Geyer CE Jr Ewer MS. Seven-year follow-up assessment of cardiac function in NSABP B-31, a randomized trial comparing doxorubicin and cyclophosphamide followed by paclitaxel (ACP) with ACP plus trastuzumab as adjuvant therapy for patients with node-positive, human epidermal growth factor receptor 2-positive breast cancer. J Clin Oncol. (2012) 30:3792–9. 10.1200/JCO.2011.40.0010 22987084 PMC3478574

[55] Dickinson JM D'Lugos AC Mahmood TN Ormsby JC Salvo L Dedmon WL. Exercise protects skeletal muscle during chronic doxorubicin administration. Med Sci Sports Exerc. (2017) 49:2394–403. 10.1249/MSS.0000000000001395 28767526

[56] Flores-Soto E Reyes-Garcia J Carbajal-Garcia A Campuzano-Gonzalez E Perusquia M Sommer B. Sex steroids effects on guinea pig airway smooth muscle tone and intracellular Ca(2+) basal levels. Mol Cell Endocrinol. (2017) 439:444–56. 10.1016/j.mce.2016.10.004 27717744

[57] Kalidhindi RSR Katragadda R Beauchamp KL Pabelick CM Prakash YS Sathish V. Androgen receptor-mediated regulation of intracellular calcium in human airway smooth muscle cells. Cell Physiol Biochem. (2019) 53:215–28. 10.33594/000000131 31299143 PMC6896987

[58] Ronnekleiv OK Zhang C Bosch MA Kelly MJ. Kisspeptin and gonadotropin-releasing hormone neuronal excitability: molecular mechanisms driven by 17beta-Estradiol. Neuroendocrinology. (2015) 102:184–93. 10.1159/000370311 25612870 PMC4459938

[59] Wang S Wang Y Qiu K Zhu J Wu Y. RCAN1 in cardiovascular diseases: molecular mechanisms and a potential therapeutic target. Mol Med. (2020) 26:118. 10.1186/s10020-020-00249-0 33267791 PMC7709393

[60] Camacho Londono JE Tian Q Hammer K Schroder L Camacho Londono J Reil JC. A background Ca2+ entry pathway mediated by TRPC1/TRPC4 is critical for development of pathological cardiac remodelling. Eur Heart J. (2015) 36:2257–66. 10.1093/eurheartj/ehv250 26069213 PMC4554959

[61] Chang AS Chang SM Garcia RL Schilling WP. Concomitant and hormonally regulated expression of trp genes in bovine aortic endothelial cells. FEBS Lett. (1997) 415:335–40. 10.1016/S0014-5793(97)01155-1 9357995

[62] Tran QK. Reciprocality between estrogen biology and calcium signaling in the cardiovascular system. Front Endocrinol (Lausanne). (2020) 11:568203. 10.3389/fendo.2020.568203 33133016 PMC7550652

[63] Norton N Necela BM Wang XL Lu T Lee HC. TRPC6 gain-of-function in doxorubicin-induced heart failure. Am Soc Hum Genet Meet. (2021) 18–22 Virtual.

[64] Chinigo G Fiorio Pla A Gkika D. TRP channels and small GTPases interplay in the main hallmarks of metastatic cancer. Front Pharmacol. (2020) 11:581455. 10.3389/fphar.2020.581455 33132914 PMC7550629

[65] Chigurupati S Venkataraman R Barrera D Naganathan A Madan M Paul L. Receptor channel TRPC6 is a key mediator of Notch-driven glioblastoma growth and invasiveness. Cancer Res. (2010) 70:418–27. 10.1158/0008-5472.CAN-09-2654 20028870

[66] Jardin I Diez-Bello R Lopez JJ Redondo PC Salido GM Smani T. TRPC6 channels are required for proliferation, migration and invasion of breast cancer cell lines by modulation of orai1 and orai3 surface exposure. Cancers (Basel). (2018) 10:331. 10.3390/cancers10090331 30223530 PMC6162527

